# Single-cell sequencing unveils mitophagy-related prognostic model for triple-negative breast cancer

**DOI:** 10.3389/fimmu.2024.1489444

**Published:** 2024-11-04

**Authors:** Peikai Ding, Shengbin Pei, Zheng Qu, Yazhe Yang, Qiang Liu, Xiangyi Kong, Zhongzhao Wang, Jing Wang, Yi Fang

**Affiliations:** Department of Breast Surgical Oncology, National Cancer Center/National Clinical Research Center for Cancer/Cancer Hospital, Chinese Academy of Medical Sciences and Peking Union Medical College, Beijing, China

**Keywords:** triple-negative breast cancer, mitophagy, single-cell sequencing, prognostic model, immunoassay

## Abstract

**Background:**

Triple-negative breast cancer (TNBC) is an aggressive subtype of breast cancer lacking hormone receptors and HER2 expression, leading to limited treatment options and poor prognosis. Mitophagy, a selective autophagy process targeting damaged mitochondria, plays a complex role in cancer progression, yet its prognostic significance in TNBC is not well understood.

**Methods:**

This study utilized single-cell RNA sequencing data from the TCGA and GEO databases to identify mitophagy-related genes (MRGs) associated with TNBC. A prognostic model was developed using univariate Cox analysis and LASSO regression. The model was validated across multiple independent cohorts, and correlations between MRG expression, immune infiltration, and drug sensitivity were explored.

**Results:**

Nine key MRGs were identified and used to stratify TNBC patients into high-risk and low-risk groups, with the high-risk group showing significantly worse survival outcomes. The model demonstrated strong predictive accuracy across various datasets. Additionally, the study revealed a correlation between higher MRG expression levels and increased immune cell infiltration, as well as potential responsiveness to specific chemotherapeutic agents.

**Conclusion:**

The mitophagy-related prognostic model offers a novel method for predicting outcomes in TNBC patients and highlights the role of mitophagy in influencing the tumor microenvironment, with potential applications in personalized treatment strategies.

## 1 Introduction

In recent times, breast cancer has established itself as the most prevalent malignant tumor in women across the globe. The Global Cancer Statistics 2022 report indicates that breast cancer ranks first among newly diagnosed cancers worldwide. It caused approximately 685,000 deaths, accounting for 6.9% of all cancer-related deaths. Despite a decline in mortality rates resulting from advances in early detection and treatment, the prevalence of breast cancer is on the rise globally, particularly in rapidly developing regions (1). Triple-negative breast cancer (TNBC) represents a highly malignant variant of the disease, accounting for approximately 10-20% of all diagnosed cases of breast cancer (2). Patients diagnosed with TNBC tend to have a less favorable prognosis, with a five-year survival rate that is considerably lower than that observed in other breast cancer subtypes. Additionally, they exhibit a higher incidence of recurrence and metastasis. The prevalence of TNBC is notably elevated among younger women and those of African descent, who also exhibit a proclivity for inferior outcomes. Consequently, there is a strong imperative to develop new prognostic models for breast cancer and to identify novel biomarkers.

Mitophagy represents a vital cellular mechanism that enables the selective degradation of damaged mitochondria, thereby maintaining mitochondrial function and cellular homeostasis. It is instrumental in cellular energy metabolism, oxidative stress responses, and programmed cell death (apoptosis) (3). More and more studies have shown that mitophagy plays a complex role in the development and progression of cancer (4). PINK1, a kinase of the mitochondria, is recruited to the outer membrane of the mitochondria and is responsible for Parkin activation. Parkin is an E3 ubiquitin ligase that facilitates the ubiquitination of damaged mitochondria, marking them for subsequent autophagic degradation (5). This mechanism plays a key role in the maintenance of mitochondrial function and the regulation of cancer cell survival as well as death. The aberrant expression levels of PINK1 and Parkin have been observed in lung and breast cancers, which points towards a potential involvement in the processes of tumorigenesis and progression (6, 7). The available evidence suggests that elevated mitophagy activity may contribute to the enhanced resilience of cancer cells to chemotherapy and radiotherapy (8). The clearance of damaged mitochondria enables cancer cells to survive the stress induced by treatment. Consequently, the inhibition of mitophagy may represent a promising avenue for enhancing the efficacy of cancer therapies.

Although TNBC is a highly invasive disease with a poor prognosis, and although mitophagy plays an essential role in cellular metabolism and apoptosis, prognostic studies targeting mitophagy-related genes in TNBC remain scarce. The majority of extant prognostic models are predicated on traditional molecular markers or gene expression profiles and thus lack a comprehensive examination of this crucial cellular process. Accordingly, the construction of a prognostic model based on mitophagy-related genes (MRGs) has the potential to address this research gap, as well as to provide new biomarkers for the personalized treatment of TNBC patients.

The advent of single-cell sequencing technology has afforded researchers a previously unattainable level of cellular resolution in the field of cancer research. This has enabled scientists to conduct in-depth investigations into the heterogeneous nature of tumors and the intricate interactions within the tumor microenvironment (9). Single-cell sequencing allows for a comprehensive analysis of gene expression, genomic variations, and epigenetic states at the individual cellular level, thereby elucidating differences among tumor cells and their responses to therapeutic interventions. In the field of malignant tumor research, single-cell sequencing has emerged as a pivotal technique for identifying novel molecular subtypes, pinpointing prospective therapeutic targets, and characterizing prognostic biomarkers.

In this study, we obtained publicly available data on triple-negative breast cancer (TNBC) from the TCGA and GEO databases. A novel prognostic model was developed through comprehensive bioinformatics analysis, utilizing nine MRGs. Patients with TNBC were stratified according to their risk profiles, resulting in the formation of two distinct groups: high-risk and low-risk. Moreover, the expression of mitophagy-related genes enabled the detection of alterations in immune infiltration and immune checkpoints in TNBC patients. The findings of our research may provide a novel perspective for the diagnosis and management of TNBC.

## 2 Methods

### 2.1 Data acquisition and preprocessing for model construction and validation in TNBC

The RNA expression profiles, gene mutation information, and clinical data for TNBC patients were sourced from the TCGA database. To build the model, the training set was utilized, while the validation set was employed to evaluate the model’s stability and accuracy. Additionally, expression profiles were retrieved from the Gene Expression Omnibus datasets GSE21653, GSE58812, and GSE65194, along with data from the Molecular Taxonomy of Breast Cancer database, to serve as external, independently validated cohorts. All datasets were formatted in TPM and log-transformed for subsequent analysis. To address potential batch effects across datasets, the “sva” package was utilized.

### 2.2 Acquisition and processing of scRNA-seq data

The single-cell RNA sequencing (scRNA-seq) dataset GSE161529, consisting of ten TNBC samples, was obtained from the Gene Expression Omnibus (GEO) database. The quality of the scRNA-seq data was evaluated using the “Seurat” and “SingleR” R packages. To maintain the integrity of the scRNA-seq data, cells with less than 10% mitochondrial gene content, more than 200 total genes, or those expressed in fewer than three cells with expression levels between 200 and 7,000 were excluded. Given the diverse sample origins, the “FindIntegrationAnchors” function from canonical correlation analysis (CCA) was utilized to eliminate any potential batch effects that could impact downstream analyses. Subsequently, the “IntegrateData” and “ScaleData” functions were used to ensure comprehensive data integration and scaling. Principal component analysis (PCA) was then employed to determine the anchors for dimensionality reduction, and the t-distributed stochastic neighbor embedding (t-SNE) method was used to examine the first 20 principal components (PCs) to identify meaningful clusters. The variability in the cell cycle among clusters was assessed using cell cycle markers integrated within the “Seurat” package.

### 2.3 Acquisition of mitophagy-related genes

MRGs were identified from the GeneCards database, with a relevance score exceeding 1.5 employed as the criterion for selecting MRGs for subsequent investigation (see **Supplementary Table**).

### 2.4 Using AUCell

The most relevant genes impacting mitophagy were identified from the single-cell RNA-sequencing data using the AUCell R package. AUCell is a computational tool that assesses the activity status of gene sets within single-cell RNA sequencing data, assigning a mitophagy activity score to each cell. The area under the curve (AUC) values for the selected MRGs were used to determine the proportion of highly expressed gene sets within each cell based on the gene expression rankings. Cells with a higher number of selected genes exhibited higher AUC values. The “AUCell explore thresholds” function was employed to identify cells actively participating in mitophagy. The cells were then categorized into two groups—high mitophagy AUC and low mitophagy AUC—based on the median AUC value. The results were graphically represented using the “ggplot2” R package.

### 2.5 Single-sample gene set enrichment analysis

ssGSEA is a frequently utilized method for calculating precise scores for enriched gene sets within a given sample. In this study, the ssGSEA method was employed to ascertain the mitophagy score (MM score) for each TNBC patient within the TCGA cohort.

### 2.6 Construction of mitophagy-related risk signatures

The process began with univariate Cox analysis to identify MRGs that had prognostic significance. These MRGs were further refined using Lasso regression, leading to the creation of a prognostic model. Each TNBC patient was then assigned a risk score based on the algorithm. The TCGA-TNBC cohort was divided into high-risk and low-risk groups according to the median value. The differences in prognosis between these two groups were then analyzed, and the model’s accuracy was evaluated.

### 2.7 Evaluation of the independence and validity of the prognostic model

To estimate the one-year, three-year, and five-year overall survival (OS) probabilities, a nomogram was constructed using risk scores, age, gender, pathological stage, and other clinical parameters as independent prognostic factors. Kaplan-Meier survival curves were generated to assess the prognostic implications, with their statistical significance evaluated using the log-rank test. The accuracy of the nomogram was further validated through calibration curves and receiver operating characteristic (ROC) curves. Additionally, a decision curve analysis (DCA) was performed to assess the net benefit of the nomogram alongside individual clinical features. A stratified analysis was also conducted to evaluate the prognostic relevance of the risk score concerning various clinical characteristics, including age, gender, clinical stage, and pathological T stage.

### 2.8 Correlation analysis of the prognostic model with tumor immunity and immunotherapy

We assessed the extent of immune infiltration in TNBC patients using data from the TCGA database, specifically from the TIMER 2.0 database, which includes results from seven distinct assessment methods. This data was utilized to generate heatmaps that illustrate the relative levels of immune cell infiltration within the tumor stroma. Following this, the genes in the prognostic risk assessment model were analyzed using ssGSEA via the GSEABase R package, which is related to immune-associated attributes. The “estimate” R package was then employed to calculate the relative abundance of stromal cells, immune cells, and tumor cells, and these values were compared across various risk categories.

### 2.9 Genomic mutation profile and drug sensitivity

The genomic mutation profiles of TNBC patients were obtained from the TCGA database and visualized using the “maftools” R package. These comprehensive gene mutation data were then integrated with the risk scores. Additionally, the “pRRophetic” R package was utilized to calculate the half-maximal inhibitory concentration (IC50) of commonly used chemotherapy drugs, allowing for an evaluation of the correlation between risk scores and drug sensitivity. The Wilcoxon signed-rank test was used to determine if there were any statistically significant differences in IC50 values between the two risk groups.

## 3 Results

### 3.1 Single-cell sequencing data analysis

**Figure 1:**
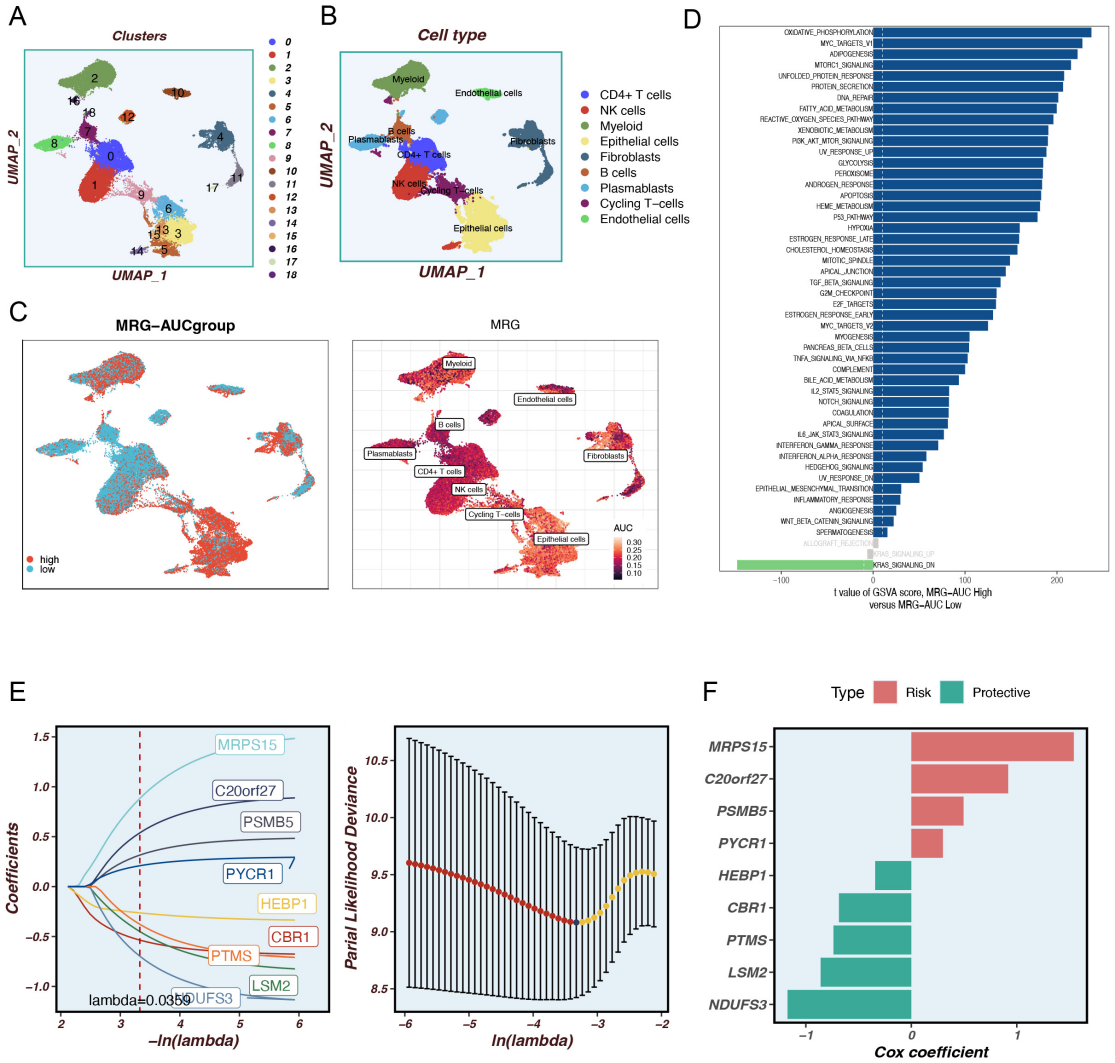
Identify differentially expressed genes and annotate cell subsets. **(A)** The tSNE plot shows the results of the dimension reduction cluster analysis. **(B)** The cells have been annotated into 9 different types of cells. **(C)** All cells were scored into high and low groups according to mitophagy-related genes (MRGs). **(D)** Analyze differentially expressed genes between high and low groups. **(E)** LASSO Cox regression analysis to develop the prognostic model. **(C)** The role of seven model genes. **(F)** The role of nine model genes.

Using dimensionality reduction algorithms (tSNE), we identified 18 distinct cell clusters characterized by different gene expression profiles (**Figure 1A**). Through cell annotation, nine different cell types were identified, including fibroblasts, myeloid cells, and tumor cells (**Figure 1B**). To explore the expression characteristics of MRGs, we evaluated the MM activity of each cell using the AUCell R package. An AUC score was attributed to all cells corresponding to the MRGs to categorize them into high- and low-AUC expression groups based on the established AUC score thresholds. Cells with elevated gene expression demonstrated elevated AUC values, a phenomenon predominantly observed in the orange-colored myeloid cells, fibroblasts, and tumor cells (**Figure 1C**). To ascertain the biological mechanisms that may be responsible for the observed differences in AUC scores, we conducted a differential expression and functional analysis. The differential expression analysis revealed that these genes were primarily associated with oxidative phosphorylation, mTORC1 signaling, fatty acid metabolism, PI3K-AKT-mTOR signaling, apoptosis, and the P53 pathway (**Figure 1D**). To further elucidate the association between MRGs and the outcome of TNBC patients, we performed an in-depth analysis of the most relevant genes affecting the activity of mitophagy obtained from single-cell data. We constructed a prognostic model using the TCGA-BC training set and identified 63 prognostic genes by univariate analysis (P < 0.01). Subsequently, we utilized LASSO Cox regression analysis to construct the prognostic model. Under the optimal regularization parameter, nine model genes were selected: MRPS5, C20orf27, PSMB5, PYCR1, HEBP1, CBR1, PTMS, LSM2, and NDUFS3 (**Figure 1E**). The calculation method for the prognostic model is as follows:


$$risk\;score=\sum_{n=i}^{k}\left(Coef_{i}\exp i\right)$$

Coefi represents the coefficient of each model gene and Expi represents the expression level of each model gene. Accordingly, a risk score was calculated for each sample and categorized into high-risk and low-risk groups. Of the nine genes used to construct the model, four were risk factors and five were protective factors (**Figure 1F**).

### 3.2 Construction and validation of the mitophagy-related prognostic model

**Figure 2:**
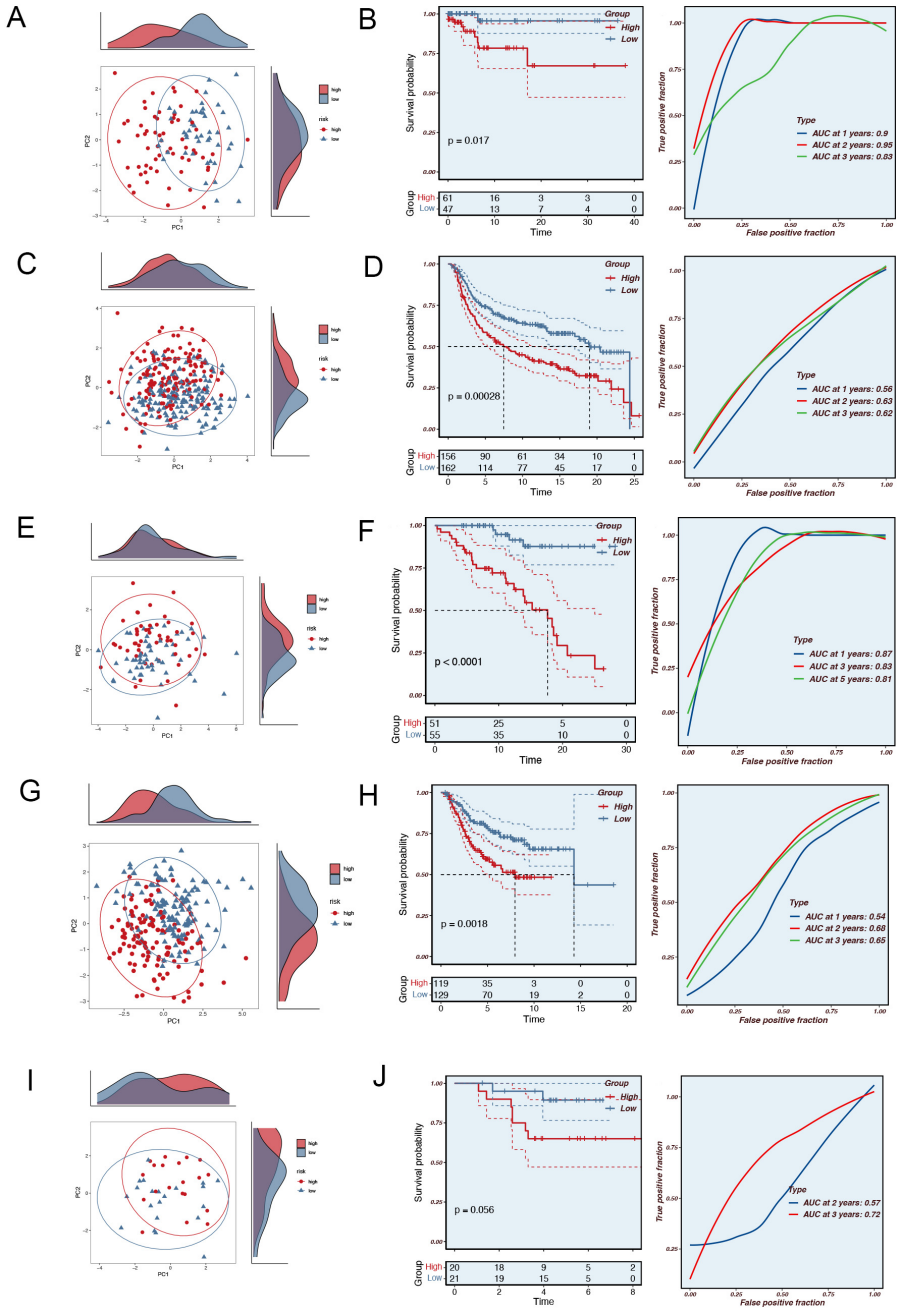
The validation of the Mitophagy-related Prognostic Model. **(A)** PCA analysis in the TCGA training set. **(B)** The area under the curve (AUC) values for the TCGA train cohort. **(C)** PCA analysis in the MetaBric validation set. **(D)** The areas under the curve at 1, 3, and 5 years for the MetaBric test group. **(E)** PCA analysis in the GSE58812 validation set. **(F)** The areas under the curve at 1, 3, and 5 years for the GSE58812 test group. **(G)** PCA analysis in the GSE2653 validation set. **(H)** The areas under the curve at 1, 3, and 5 years for the GSE2653 test group. **(I)** PCA analysis in the GSE65194 validation set. **(J)** The areas under the curve at 1, 3, and 5 years for the GSE65194 test group.

**Figure 3:**
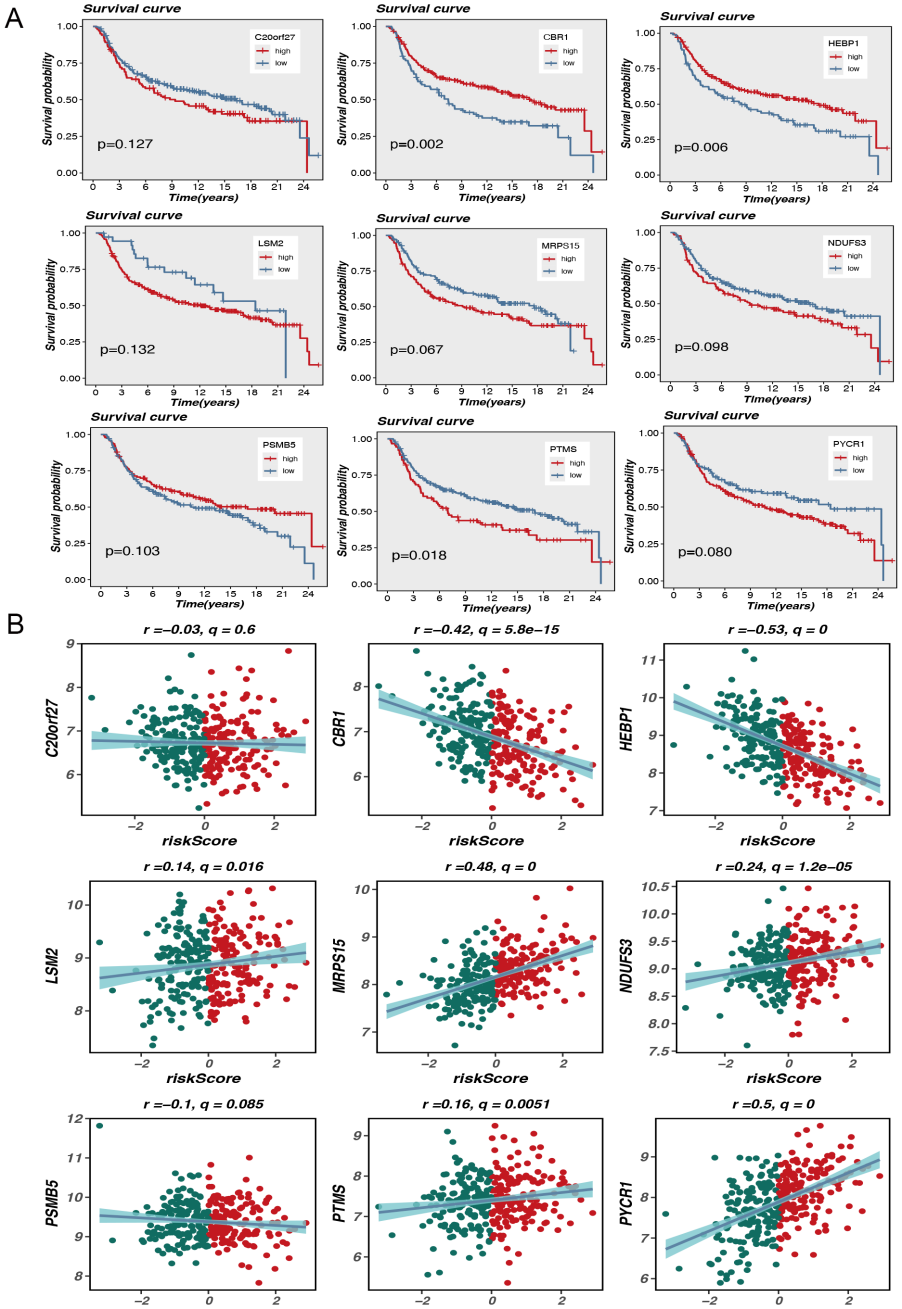
Model gene survival and risk assessment. **(A)** Model gene survival analysis. **(B)** Model genes correlate with risk scores.

Principal component analysis (PCA) of the nine model genes across the TCGA, MetaBric, GSE58812, GSE2653, and GSE65194 datasets revealed that the model effectively stratified TNBC patients into two risk groups. To ascertain the precision of the MRGs in prognosticating the outcome of TNBC patients, we conducted a receiver operating characteristic (ROC) curve analysis on both the training and testing datasets. In the training set TCGA database, TNBC patients can be effectively categorized into two risk groups (**Figure 2A**). Meanwhile, there was a significant difference in the prognosis of patients in the two groups (P=0.0017, **Figure 2B**). In the MetaBric test set, it was possible to distinguish well between the two groups of patients while there was a significant difference in prognosis between the two groups (P=0.00028, **Figures 2C, D**). In the GSE58812 test set, the AUC was consistently above 0.8, indicating the high accuracy of the model in assessing patient prognosis (**Figures 2E, F**). In the GSE2653 test set, PCA did a good job of separating patients into high-risk and low-risk groups (**Figure 2G**), and a similar pattern was observed for both patient survival and AUC (**Figure 2H**). Although the PCA in the GSE65194 cohort effectively stratified patients into high- and low-risk groups (**Figure 2I**), and a trend of poorer prognosis was observed in the high-risk group, the difference in prognosis between the two groups did not reach statistical significance (P=0.056, **Figure 3J**). This result suggests that the relatively small sample size or cohort-specific variability may have limited the ability to detect a statistically significant difference. Further validation with larger cohorts may help confirm these findings. In conclusion, the prognostic model based on MRGs demonstrated high predictive accuracy in all four independent cohorts.

### 3.3 Prognostic value of mitophagy-related genes in triple-negative breast cancer: correlations with survival and risk scores

The aim of this research is to analyze the potential association of expression levels of nine MRGs with survival outcomes and risk scores in TNBC patients. Kaplan-Meier survival analysis showed a difference in overall outcome between individuals with high and low expression of these genes. In particular, elevated expression of genes such as CBR1, HEBP1, and PTMS was markedly linked to diminished survival rates, with P-values of 0.002, 0.006, and 0.018, respectively. This suggests that these genes may serve as crucial prognostic predictors for TNBC. Although the P-values for some genes, such as MRPS15 and PYCR1, were slightly above the traditional significance level (p<0.05), they still exhibited a general trend associated with poor prognosis (**Figure 3A**).

In addition, scatterplot analysis showed the association between the expression level of each gene and the risk score. Expression of CBR1 and HEBP1 was significantly negatively correlated with risk scores, with Pearson correlation coefficients of -0.42 (q = 5.8e-15) and -0.53 (q = 0), respectively. This indicates that high level of these gene expression may be associated with lower risk scores, further supporting their potential protective role in prognosis. Conversely, the expression of LSM2 and MRPS15 showed a positive correlation with risk scores, with correlation coefficients of 0.14 (q = 0.016) and 0.48 (q = 0), respectively. These results indicate that high expression levels of these genes may be linked to increased risk scores and poorer prognosis (**Figure 3B**).

The results indicate that the analysis of mitophagy-related gene expression patterns may serve as an effective stratification method for TNBC patients into distinct risk groups and for predicting their survival outcomes. The findings suggest that these genes may be critical in the progression of TNBC and provide new potential targets for personalized therapy.

### 3.4 Construction of a nomogram model and drug sensitivity analysis

**Figure 4:**
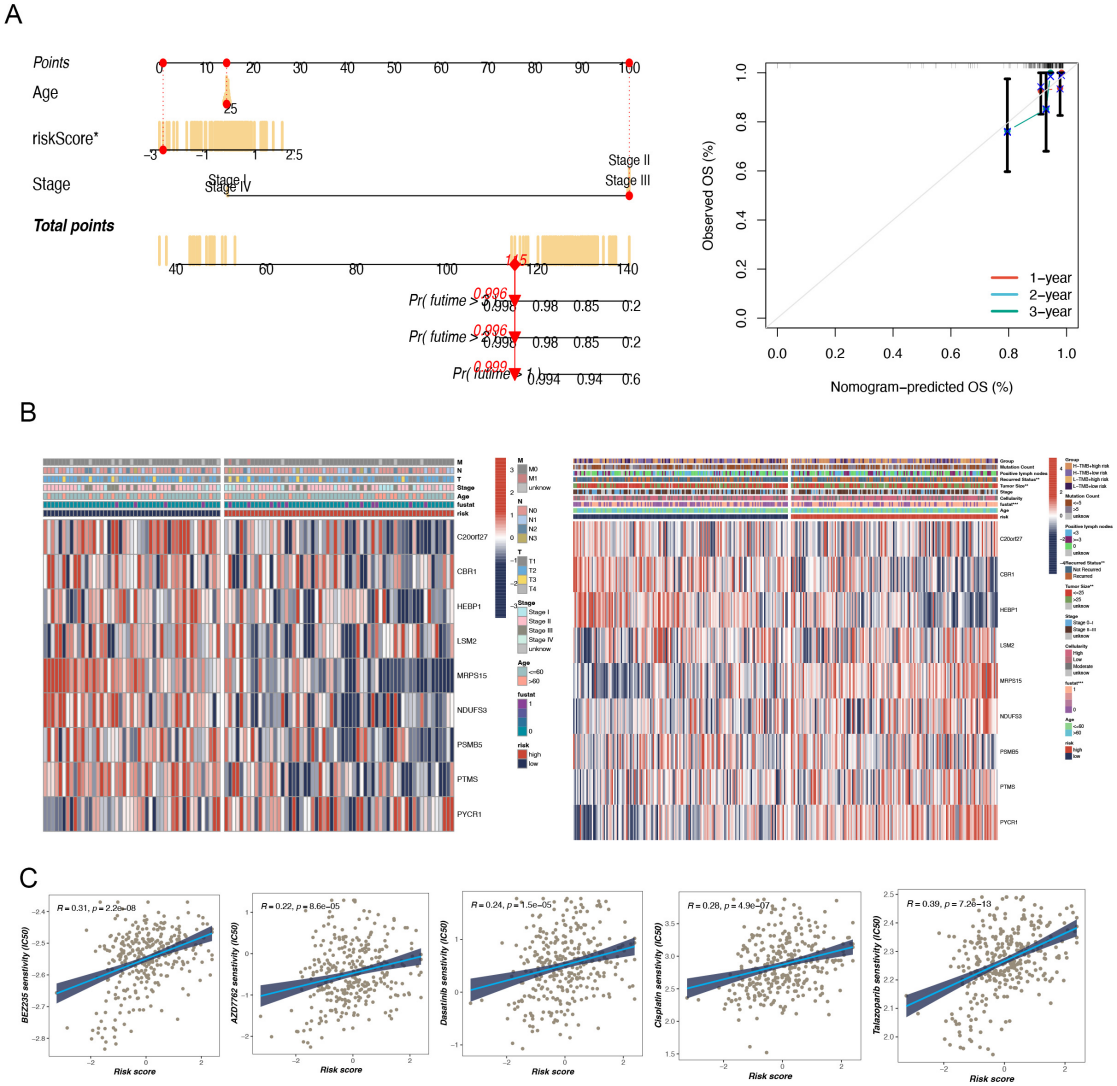
The construction of a nomogram and clinical correlation analysis. **(A)** Nomogram to assess the risk of BC patients. **(B)** There were significant differences in tumor size and recurrence status between high and low-risk groups. **(C)** Potential drug screening in high-risk patients.

Based on the TCGA data, we selected several key variables (such as risk score, age, and cancer stage) and constructed a nomogram model to more precisely quantify the risk for breast cancer patients (**Figure 4A**). As an intuitive and practical tool, the nomogram plays an important role in cancer prognosis prediction and personalized treatment (10). By integrating multiple predictive variables, the nomogram provides accurate assessments of survival probability and recurrence risk, thereby aiding clinicians in making more optimized treatment decisions, which in turn enhances patient outcomes and quality of life. To ascertain the veracity and predictive efficacy of the nomogram model, we conducted a calibration curve analysis, which demonstrated that the predicted values of the model were highly consistent with observed values at different time points, indicating high predictive accuracy.

Furthermore, a clinical heatmap was generated using the prognostic model genes to evaluate the associations among the model genes and clinicopathologic features (such as lymph node positivity, recurrence status, tumor size, etc.) (**Figure 4B**). The results showed significant differences between the two groups, especially in terms of tumor size and recurrence status (p < 0.05).

A notable meaningful positivity was detected between multidrug sensitivity (IC50) and TNBC risk score. This suggests that an elevated risk score is indicative of heightened sensitivity among patients to these pharmaceutical agents. The drugs in question are Dasatinib, AP-24534, AZD7762, Cisplatin, BEZ235, and Talazoparib (**Figure 4C**). These findings are of considerable clinical significance. Patients with higher risk scores may exhibit greater sensitivity to these drugs, thereby warranting their prioritization in the treatment regimen. Of the drugs under consideration, talazoparib demonstrated the highest correlation (R = 0.39), indicating its potential efficacy in high-risk TNBC patients.

### 3.5 Impact of tumor mutational burden: a comprehensive study of risk scores and survival analysis

**Figure 5:**
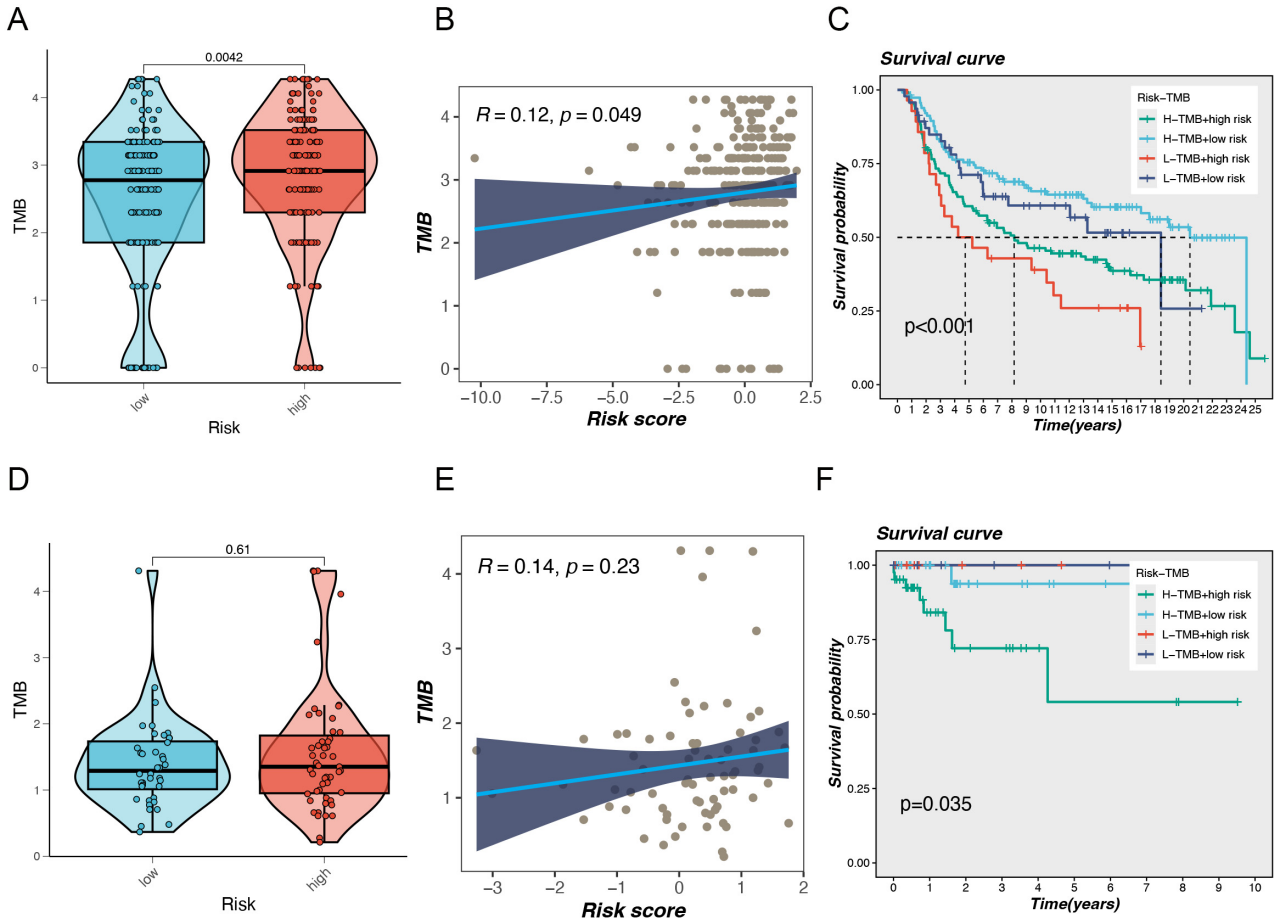
Gene mutation analysis. **(A)** Differences in Tumor Mutational Burden (TMB) levels between the two risk groups in the MetaBric database. **(B)** The correlation between TMB and risk score. **(C)** Correlation analysis between TMB and prognosis. **(D)** Differences in Tumor Mutational Burden (TMB) levels between the two risk groups in the TCGA database. **(E)** The correlation between TMB and risk score. **(F)** Correlation analysis between TMB and prognosis.

TMB is a biomarker that has been widely accepted as an essential factor in cancer research and treatment, particularly in the setting of immunotherapy. This metric is increasingly being acknowledged as a potential indicator of responsiveness to immune checkpoint inhibitors (ICIs) (11). A high TMB is associated with an elevated number of mutations, which has the potential to generate an enhanced generation of neoantigens that can be recognized by the immune system, thereby improving ICIs’ efficacy (12). Our study found that in the MetaBric database, the high-risk group exhibited a higher mutation frequency, which was statistically significant (P = 0.0042, **Figure 5A**). Correlation analysis revealed a positive relationship between risk score and mutation frequency, with higher risk scores associated with higher mutation frequencies (**Figure 5B**). Previous studies have shown that tumors with a high mutational burden generally have a worse prognosis. This view has also been confirmed in breast cancer research. A study by Barroso-Sousa et al. found that high TMB is not only common in breast cancer, especially TNBC, but is also associated with worse survival (13). Our results provide further evidence to support the hypothesis that high mutational load may serve as a negative prognostic indicator. In terms of survival analysis, the low-risk and high-TMB groups had the most favorable prognosis for survival, whereas the high-risk and low-TMB groups had the worst prognosis, with a statistically significant difference between them (**Figure 5C**). Similar prognostic differences were observed in the TCGA database, although no statistical difference was found between TMB and risk rating in this database (**Figures 5D–F**).

### 3.6 Analysis of tumor microenvironment and immune cell infiltration

**Figure 6:**
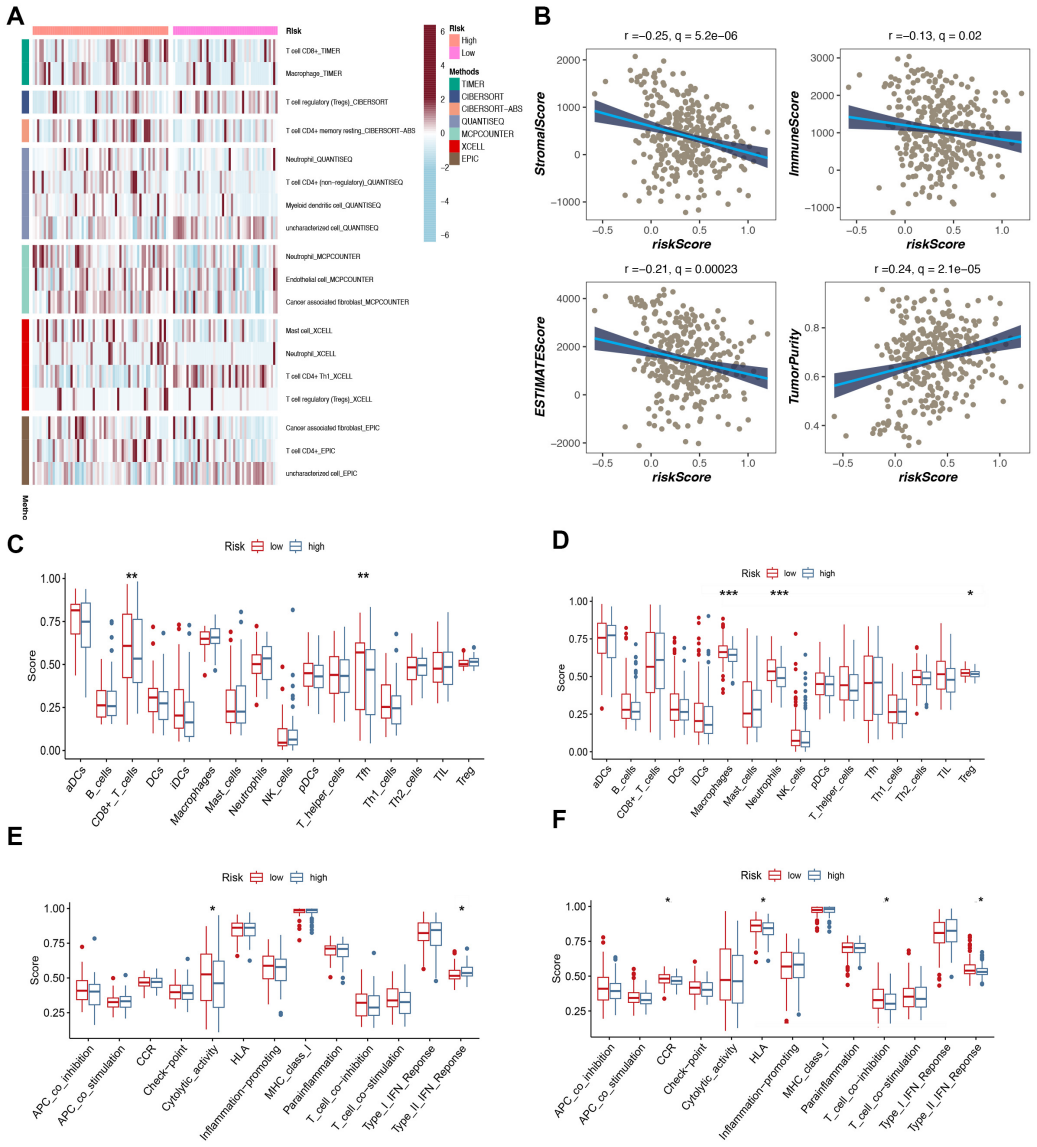
Analysis of immune microenvironment. **(A)** The distribution and association of tumor-infiltrating immune cells (TICs) in two risk groups. **(B)** Correlation analysis of immune score and risk score, ESTIMATE score and risk score, Stromal score and risk score, tumor purity and risk score. **(C, D)** Analysis of immune cell infiltration. **(E, F)** Analysis of the immunization process.

The level of immune cell infiltration in each test specimen was assessed according to seven different methods to gain a deeper understanding of the distribution and correlation of 18 types of tumor-infiltrating immune cells (TICs) in the TCGA-BC cohort. The high-risk group showed increased levels of immune cell infiltration compared to the low-hazard group (**Figure 6A**). In particular, the high-risk group showed increased amounts of immune cell infiltration, particularly macrophages, neutrophils, and cancer-associated fibroblasts. The low-risk group had significantly enhanced stromal, immune, and ESTIMATE scores, suggesting increased general immune status and the immunogenicity of the tumor microenvironment (TME) (P < 0.05). Additionally, a positive correlation with tumor purity was observed (**Figure 6B**). The expression levels of 29 immune cells were further analyzed in the training and validation sets, and the box plot results demonstrated that two types of cells in the training set exhibited high infiltration in the high-risk group: CD8+ T cells and Tfh (**Figure 6C**). The cytolytic activity represents a crucial mechanism through which the immune system can control and eliminate harmful cells within the body. Our findings revealed elevated cytolytic activity in the high-risk group (**Figure 6E**). In the validation set (Metabric), macrophages, neutrophils, and Treg exhibited high infiltration in the high-risk group (**Figure 6D**). Additionally, they demonstrated heightened activity in CCR, HLA, and other biological processes (**Figure 6F**).

### 3.7 Insights into immune therapy efficacy through checkpoint analysis

**Figure 7:**
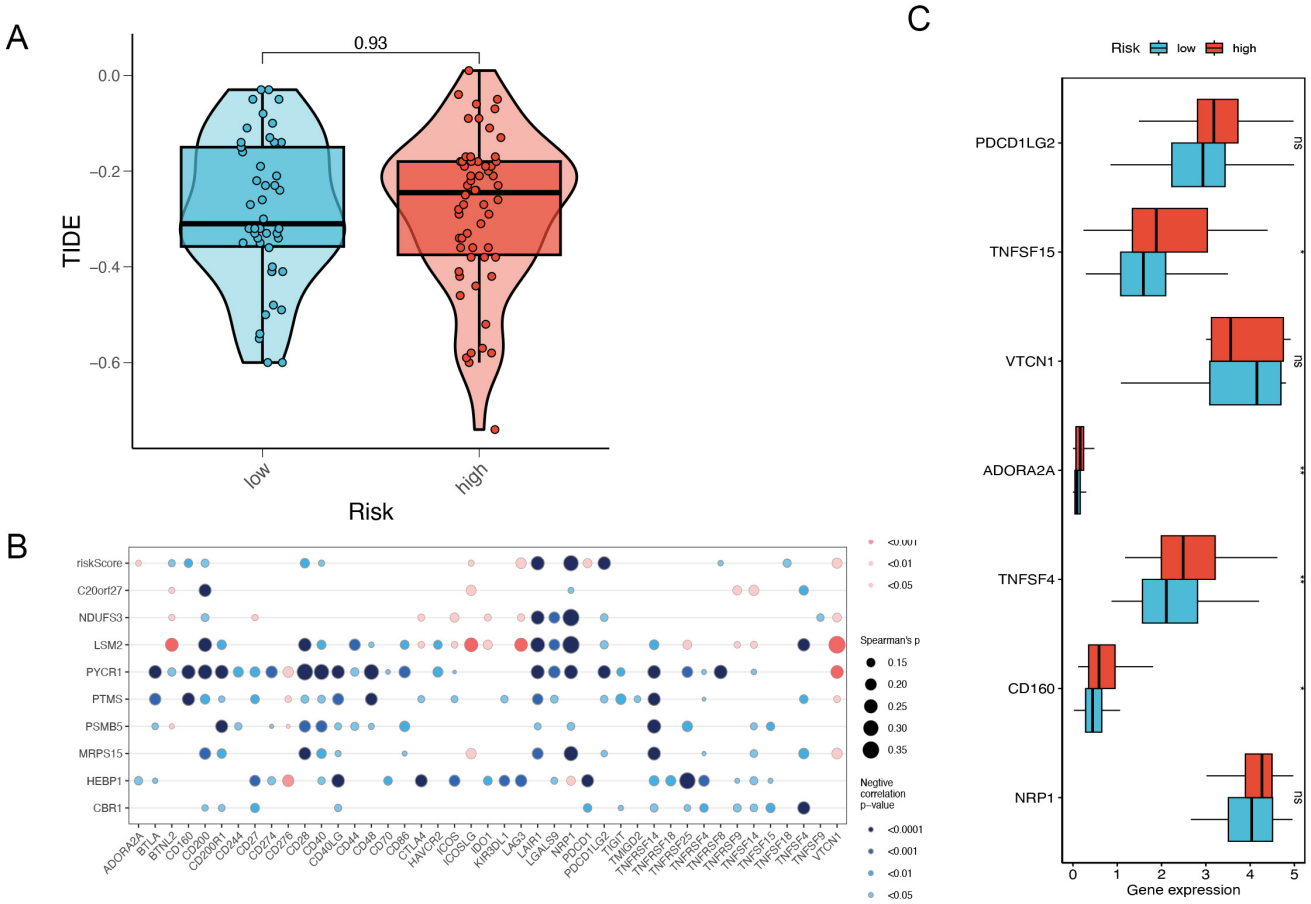
Risk gene and immune checkpoint analysis. **(A)** The difference in TIDE scores between high and low-risk groups. **(B)** Risk gene and immune checkpoint correlation. **(C)** Differences in the abundance of immune-checkpoint-related genes between high and low-risk groups. *P<0.05, **P< 0.01, ***P<0.001, ns indicates No significance.

Tumors have two distinct immune escape mechanisms: on the one hand, some immunosuppressive factors prevent T-cell infiltration; on the other hand, some tumors are functionally inactivated despite high levels of cytotoxic T-cell infiltration. TIDE (Tumor Immune Dysfunction and Exclusion) is a computational tool for evaluating the tumor immune escape mechanism and a computational tool for predicting immune checkpoint inhibitor (ICI) treatment response. Tumor immune escape is predicted by a combined assessment of the activity of these two mechanisms. Higher TIDE scores were associated with poorer immune checkpoint inhibition therapy. The results of the violin plot showed higher TIDE scores in the high-risk group than in the low-risk group, which was consistent but not statistically significant and may be related to the small sample size (**Figure 7A**). Meanwhile, the bubble plot showed the model genes correlated with immune checkpoints, in which PYCR1 was significantly negatively correlated with immune checkpoints (**Figure 7B**). We further analyzed the expression levels of immune checkpoints in high and low-risk groups, in which TNFSF15, ADORA2A, TNFSF4 and CD160 were expressed at higher levels in high-risk groups (**Figure 7C**).

### 3.8 Differential pathway enrichment in high- and low-risk TNBC patients: insights from GSEA and GSVA analysis

**Figure 8:**
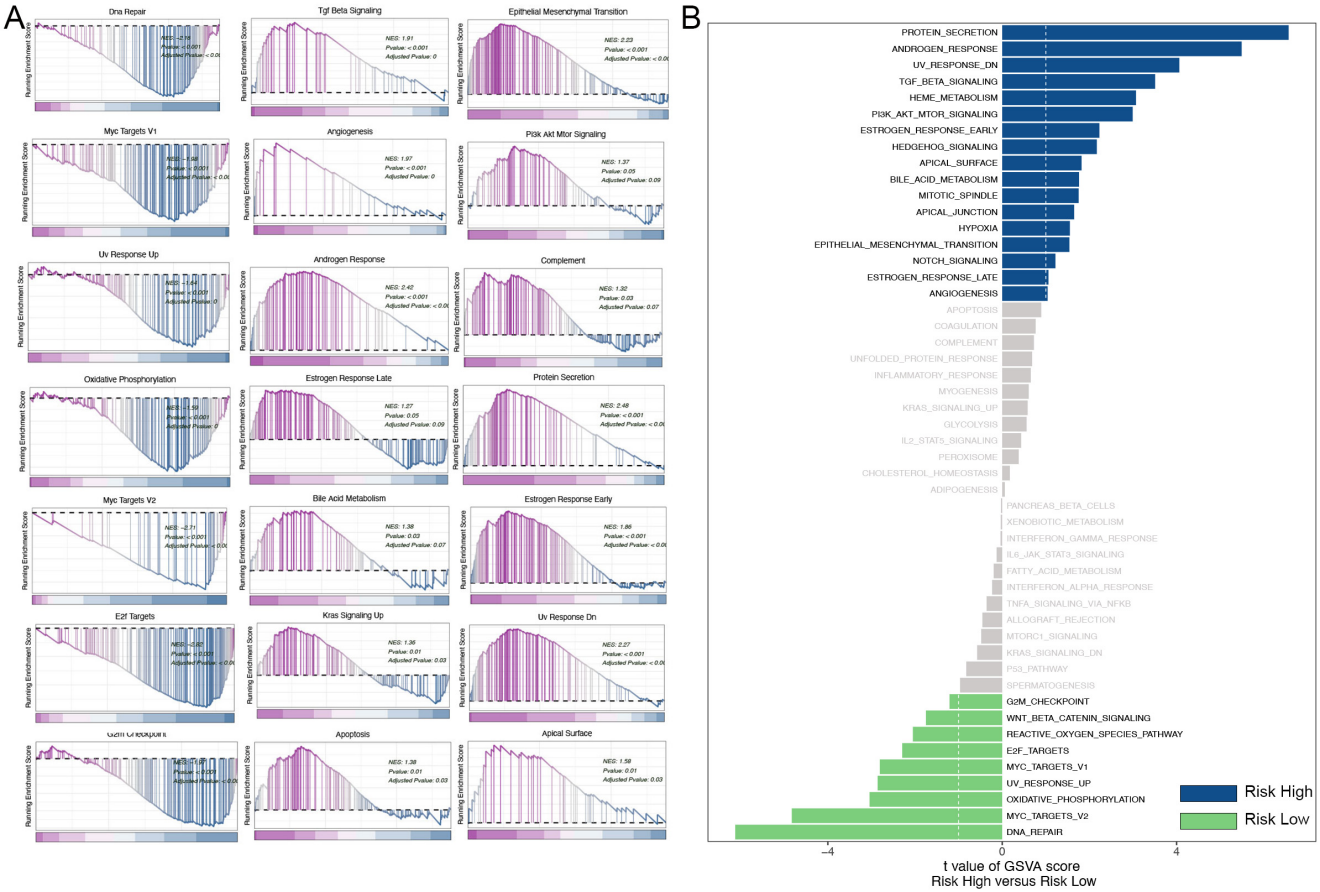
Analysis of GSEA and GSVA. **(A)** GSEA pathway enrichment analysis. **(B)** GSVA pathway enrichment analysis.

Gene Set Enrichment Analysis (GSEA) was employed to categorize TNBC patients into high-risk and low-risk groups based on the risk model constructed from mitophagy-related genes. The results demonstrated notable discrepancies in numerous signaling pathways between the two groups. The high-risk group exhibited enrichment of the TGF-β signaling pathway. The transforming growth factor-beta (TGF-β) signaling pathway plays a pivotal role in regulating a multitude of cellular processes, including proliferation, differentiation, and apoptosis. Additionally, it plays a pivotal role in the tumor microenvironment, influencing processes such as tumor cell proliferation, immune evasion, and metastasis (14). The upregulation of the TGF-β signaling pathway in the high-risk group may facilitate tumor cell invasion and metastasis while simultaneously impeding the immune system’s anti-tumor response. The high-risk group exhibited an enrichment of epithelial-mesenchymal transition (EMT), which suggests an elevated propensity for tumor cell invasion and metastasis. Epithelial-mesenchymal transition (EMT) is a process by which epithelial cells gain a mesenchymal phenotype, a transformation that is closely associated with cancer metastasis (15). The PI3K/AKT/mTOR signaling pathway, which plays a pivotal role in cell growth, proliferation, and survival, was also found to be upregulated in the high-risk group. The aberrant activation of the PI3K/AKT/mTOR pathway is a common occurrence in a multitude of cancers, and it serves to promote tumor growth and drug resistance (16). The observed upregulation of this pathway in the high-risk group may contribute to the rapid proliferation of tumor cells and their anti-apoptotic capabilities, thereby increasing tumor aggressiveness and resistance to therapy. In contrast, the DNA repair pathway was found to be more active in the low-risk group, indicating that the DNA repair capacity may be compromised in the high-risk group, which could result in increased genomic instability. The results demonstrate a notable enrichment of multiple critical biological pathways in triple-negative breast cancer. Among these pathways are those associated with cell cycle regulation, genomic stability, cellular metabolism, tumor invasion, and metastasis (**Figure 8A**). To validate these results, we conducted further analysis using gene set variation analysis (GSVA). The results of GSVA were also consistent, with the high-risk group being enriched in pathways such as the TGF-β signaling, PI3K/AKT/mTOR signaling, and others, further supporting the conclusions drawn by GSEA (**Figure 8B**).

## 4 Discussion

TNBC is distinguished by its high degree of heterogeneity and intricate molecular composition, which collectively contribute to its generally poor prognosis. Conventional classification techniques frequently prove inadequate for encompassing the full spectrum of TNBC, resulting in considerable obstacles in clinical management and a significant burden on patients and society alike. The heterogeneous nature of TNBC is evident not only in its molecular characteristics, but also in the significant variability observed in its clinical presentation and response to treatment (17). The current classification methods are inadequate for capturing the full spectrum of molecular diversity observed in TNBC, leading to considerable uncertainty in patient prognosis. Consequently, reliance on a single classification standard is inadequate for the effective prediction of TNBC treatment response and survival outcomes. In light of these considerations, we proposed a novel perspective based on the analysis of mitophagy-related gene expression as a means of evaluating TNBC prognosis, thereby providing a more reliable foundation for personalized treatment. This approach not only enhances patient prognosis but also offers guidance for the development of novel therapeutic strategies.

Mitophagy is a selective form of autophagy, a process by which cells degrade mitochondria. It is a vital mechanism for maintaining cellular health and function, playing a pivotal role in processes such as cellular stress, aging, and cancer (18). Mitophagy has been demonstrated to play a complex and dual role in cancer, with the potential to either promote cancer cell survival or to inhibit cancer initiation and progression (4). Despite the growing body of research on mitophagy in breast cancer in recent years, with notable advances in TNBC, the majority of these studies remain confined to fundamental mechanistic investigation (19). The precise role of mitophagy in TNBC and its clinical applications remain to be fully elucidated through rigorous scientific inquiry. Notably, there is a significant deficit in the construction of prognostic models based on mitophagy. This is the first instance in which a prognostic model for TNBC has been constructed using mitophagy-related genes. Our study not only addresses this gap in the literature but also establishes a foundation for further, more in-depth research in the future.

The study employed single-cell sequencing to examine the function of MRGs in TNBC. Through Cox and Lasso regression analyses, nine MRGs were identified and used to construct a TNBC prognostic model. This model was validated in four independent datasets (METABRIC, GSE58812, GSE2653, and GSE65194) and exhibited strong predictive accuracy and robustness. While current prognostic models like MammaPrint^®^, Breast Cancer Index (BCI), and Oncotype DX are effective for certain breast cancer subtypes, they lack optimization for TNBC. As a result, their predictions for TNBC patients are not as reliable (20–22). Existing TNBC-specific models frequently fail to consider the pivotal function of mitophagy, which is essential for cellular metabolism, stress response, and tumor survival. A prognostic model based on MRGs thus offers greater biological and clinical relevance. Our study represents an innovative application of single-cell sequencing, which has been instrumental in uncovering the heterogeneity of TNBC and in the development of a more precise prognostic model. Our findings indicate that patients classified as high-risk by our model are more likely to develop larger tumors and experience recurrence, thereby demonstrating the model’s utility in predicting outcomes in TNBC.

TNBC is characterized by the absence of specific hormone receptors and HER2 expression, which restricts the range of available treatment options and renders chemotherapy the most commonly employed systemic therapy. To further validate the clinical utility of our model, we conducted an analysis of chemotherapy drugs. Five drugs were identified as particularly effective in the high-risk group: BEZ235, AZD7762, dasatinib, cisplatin, and talazoparib. AZD7762, a Chk1 inhibitor, has demonstrated considerable promise in the treatment of breast cancer, particularly in cases where p53 mutations and TNBC are present. This is due to its ability to enhance the efficacy of radiotherapy and chemotherapy (23, 24). Dasatinib has also been shown to possess notable therapeutic potential in the treatment of breast cancer, particularly in the TNBC and HER2-positive subtypes. This is achieved by targeting breast cancer stem cells and enhancing the sensitivity of cancer cells to chemotherapy (25, 26). Cisplatin, a traditional platinum-based chemotherapy drug, has demonstrated efficacy against TNBC by inhibiting the EMT process. Furthermore, it has been shown to enhance the efficacy of other drugs, such as paclitaxel, in treating TNBC (27). Talazoparib, a recently developed PARP inhibitor, has demonstrated remarkable efficacy in patients with HER2-negative advanced breast cancer and BRCA mutations. This treatment has demonstrated marked improvements in both OS and PFS, as well as quality of life (28, 29). Therefore, Talazoparib may become a crucial element in personalized treatment plans for high-risk patients. Clinicians may consider integrating Talazoparib into treatment regimens based on the patient’s BRCA mutation status, to optimize therapeutic outcomes while reducing adverse effects. For other drugs (such as dasatinib, AZD7762, and cisplatin), it is recommended that clinicians personalize drug selection based on the patient’s molecular characteristics (such as gene mutations or expression profiles).

In cancer research, immune infiltration is a key concept as it involves the immune system’s response to tumors or lesions. The degree and type of immune infiltration can significantly influence disease progression, patient prognosis, and treatment response (30). In recent years, immunotherapy, particularly immune checkpoint inhibitors, has made remarkable progress in various types of tumors (31). However, triple-negative breast cancer (TNBC), due to its high aggressiveness and lack of effective targeted therapies, often does not respond well to traditional treatments. In this context, immunotherapy has become a critical research focus for TNBC treatment. Currently, the application of immunotherapy in TNBC has shown some efficacy. Clinical trials such as KEYNOTE-355 and IMpassion130 have found that PD-L1-positive TNBC patients respond better to immune checkpoint inhibitors combined with chemotherapy, significantly prolonging PFS and OS (32, 33). However, the efficacy of immunotherapy varies among individuals, and not all patients benefit (34). Additionally, the occurrence of immune-related adverse events presents a significant challenge, requiring further optimization of treatment strategies.

In this study, we found that the infiltration levels of cancer-associated fibroblasts (CAFs) and endothelial cells were significantly higher in the high-risk group compared to the low-risk group. This finding is consistent with existing literature and further validates the critical role these cells play in tumor progression and patient prognosis. Specifically, CAFs not only promote tumor growth and metastasis by secreting growth factors and cytokines, but they also interact with immune cells, altering the tumor microenvironment and thereby enhancing the malignancy of the tumor (34). Additionally, endothelial cells significantly facilitate breast cancer cell invasion and metastasis through metabolic reprogramming and the secretion of specific factors (35). These mechanisms may elucidate the association between higher endothelial cell infiltration in the high-risk group and a poorer prognosis. Furthermore, we conducted a comprehensive investigation into the composition of immune cells, immune infiltration, and immune scoring in TNBC. These studies provide crucial theoretical support and potential therapeutic targets for immunotherapy in TNBC. These findings not only enhance our comprehension of the immune microenvironment in TNBC but also provide direction for prospective treatment strategies.

This study employed TIDE analysis to further elucidate the impact of immune escape mechanisms on immunotherapy in high-risk TNBC patients. Although the TIDE scores were higher in the high-risk group, indicating that these patients may respond poorly to immune checkpoint inhibitors (ICI), the difference in TIDE scores was not statistically significant, likely due to the small sample size or data heterogeneity. Nevertheless, the observed trend in TIDE scores suggests that immune escape mechanisms may be more active in high-risk patients. It is recommended that future research should aim to increase the sample size or incorporate additional immune marker analysis to improve the ability to predict responses to immunotherapy. Furthermore, the model gene PYCR1 was found to be negatively correlated with immune checkpoints, indicating that PYCR1 may play a pivotal role in immune escape. The elevated expression of immune checkpoints, including TNFSF15, ADORA2A, TNFSF4, and CD160, in the high-risk group indicates that these checkpoints may represent promising therapeutic targets in the future.

While this study makes a notable contribution to the understanding of the immune microenvironment in TNBC, it is not without limitations. First, due to the limitations of the experimental design, we were unable to conduct *in vivo* and *in vitro* experiments to validate these findings. Instead, we relied on data analysis. Although our results were validated using multiple external datasets, the lack of our dataset may limit the generalizability of the findings. It is therefore recommended that future research should include experimental validation to solidify these findings and explore more precise immunotherapy strategies.

## 5 Conclusion

This study developed a mitophagy-related prognostic model for TNBC using single-cell sequencing data, effectively stratifying patients into high- and low-risk groups. Patients at high risk exhibited diminished survival, increased tumor size, and elevated recurrence rates. A drug sensitivity analysis identified chemotherapeutic agents, including talazoparib, as potentially more effective in high-risk patients. The immune analysis revealed an increased infiltration of macrophages, neutrophils, and cancer-associated fibroblasts in patients at high risk, which has been linked to tumor progression. The correlation between PYCR1 and immune checkpoints indicates its involvement in immune evasion, thereby offering potential targets for immunotherapy. The model offers insights into TNBC prognosis and provides a foundation for personalized treatment strategies.

## Data Availability

The original contributions presented in the study are included in the article/**Supplementary Material**. Further inquiries can be directed to the corresponding authors.
